# Multimodal Imaging in Epilepsy Surgery for Personalized Neurosurgical Planning

**DOI:** 10.3390/jpm15120601

**Published:** 2025-12-05

**Authors:** Joaquin Fiallo Arroyo, Jose E. Leon-Rojas

**Affiliations:** 1 NeurALL Research Group, Quito 170157, Ecuador; joaquin.fiallo@udla.edu.ec; 2 Cerebro, Emoción y Conduca (CEC) Research Group, Escuela de Medicina, Universidad de las Américas (UDLA), Quito 170124, Ecuador

**Keywords:** epilepsy, epilepsy surgery, multimodal imaging, drug-resistant epilepsy, epilepsy imaging

## Abstract

Drug-resistant epilepsy affects nearly one-third of individuals with epilepsy and remains a major cause of neurological morbidity worldwide. Surgical intervention offers a potential cure, but its success critically depends on the precise identification of the epileptogenic zone and the preservation of eloquent cortical and subcortical regions. This review aims to provide a comprehensive synthesis of current evidence on the role of multimodal neuroimaging in the personalized presurgical evaluation and planning of epilepsy surgery. We analyze how structural, functional, metabolic, and electro-physiological imaging modalities contribute synergistically to improving localization accuracy and surgical outcomes. Structural MRI remains the cornerstone of presurgical assessment, with advanced sequences, post-processing techniques, and ultra-high-field (7 T) MRI enhancing lesion detection in previously MRI-negative cases. Functional and metabolic imaging, including FDG-PET, ictal/interictal SPECT, and arterial spin labeling MRI, offer complementary insights by revealing regions of altered metabolism or perfusion associated with seizure onset. Functional MRI enables non-invasive mapping of language, memory, and motor networks, while diffusion tensor imaging and tractography delineate critical white-matter pathways to minimize postoperative deficits. Electrophysiological integration through EEG source imaging and magnetoencephalography refines localization when combined with MRI and PET data, forming the basis of multimodal image integration platforms used for surgical navigation. Our review also briefly explores emerging intraoperative applications such as augmented and virtual reality, intraoperative MRI, and laser interstitial thermal therapy, as well as advances driven by artificial intelligence, such as automated lesion detection and predictive modeling of surgical outcomes. By consolidating recent developments and clinical evidence, this review underscores how multimodal imaging transforms epilepsy surgery from a lesion-centered to a patient-centered discipline. The purpose is to highlight best practices, identify evidence gaps, and outline future directions toward precision-guided, minimally invasive, and function-preserving neurosurgical strategies for patients with drug-resistant focal epilepsy.

## 1. Introduction

Drug-resistant epilepsy is a significant clinical challenge, affecting roughly 30% of epilepsy patients who do not respond to medications [1]. For these individuals, epilepsy surgery can be a curative or palliative option, especially when a focal epileptogenic zone can be identified and safely resected. The success of epilepsy surgery heavily depends on accurately localizing the epileptogenic zone while preserving essential brain functions; neuroimaging plays a pivotal role in this process, enabling a personalized surgical approach tailored to each patient’s unique brain anatomy and pathology [1,2]. Indeed, the presence of a detectable lesion on imaging is one of the strongest predictors of postoperative seizure freedom [2]. A meta-analysis of 40 studies found that patients with an identifiable lesion on magnetic resonance imaging (MRI) or histopathology had 2.5–3 times higher odds of achieving seizure freedom after surgery compared to those without a lesion [3]. Conversely, patients with “MRI-negative” epilepsy (no lesion seen on conventional MRI) tend to have lower success rates and often require more extensive evaluation [4]. This underscores the need for advanced imaging techniques to uncover subtle abnormalities and guide surgery in a personalized manner.

Modern epilepsy surgery planning is a multidisciplinary endeavor involving neurologists, neurosurgeons, neuroradiologists, neuropsychologists and other specialists. Multimodal imaging data, structural, functional, metabolic, and electrophysiological, are integrated to formulate an optimal, patient-specific surgical plan [5]. Surgical treatment of drug-resistant epilepsy encompasses several established and emerging neurosurgical techniques that aim to remove, disconnect, or modulate the epileptogenic network [1,2,3,4,5,6]. The most frequently performed procedures include anterior temporal lobectomy and selective amygdalohippocampectomy for mesial temporal lobe epilepsy, which remain the gold-standard interventions with the highest rates of long-term seizure freedom [1,2,3,4,5,6]. In cases of neocortical epilepsy, lesionectomy or focal cortical resection is performed when a discrete lesion can be localized, while multiple subpial transections may be used in eloquent cortical areas where complete excision would cause deficits [1,2,3,4,5,6]. For patients unsuitable for resective surgery, disconnective procedures such as corpus callosotomy or hemispherotomy can reduce seizure propagation. Additionally, neuromodulatory approaches including vagus nerve stimulation (VNS), deep brain stimulation (DBS), and responsive neurostimulation (RNS) have become important adjunctive options for patients with multifocal or non-localizable epilepsy. Together, these surgical modalities form a continuum of personalized interventions aimed at maximizing seizure control while preserving neurological function [1,2,3,4,5,6].

**Figure 1:**
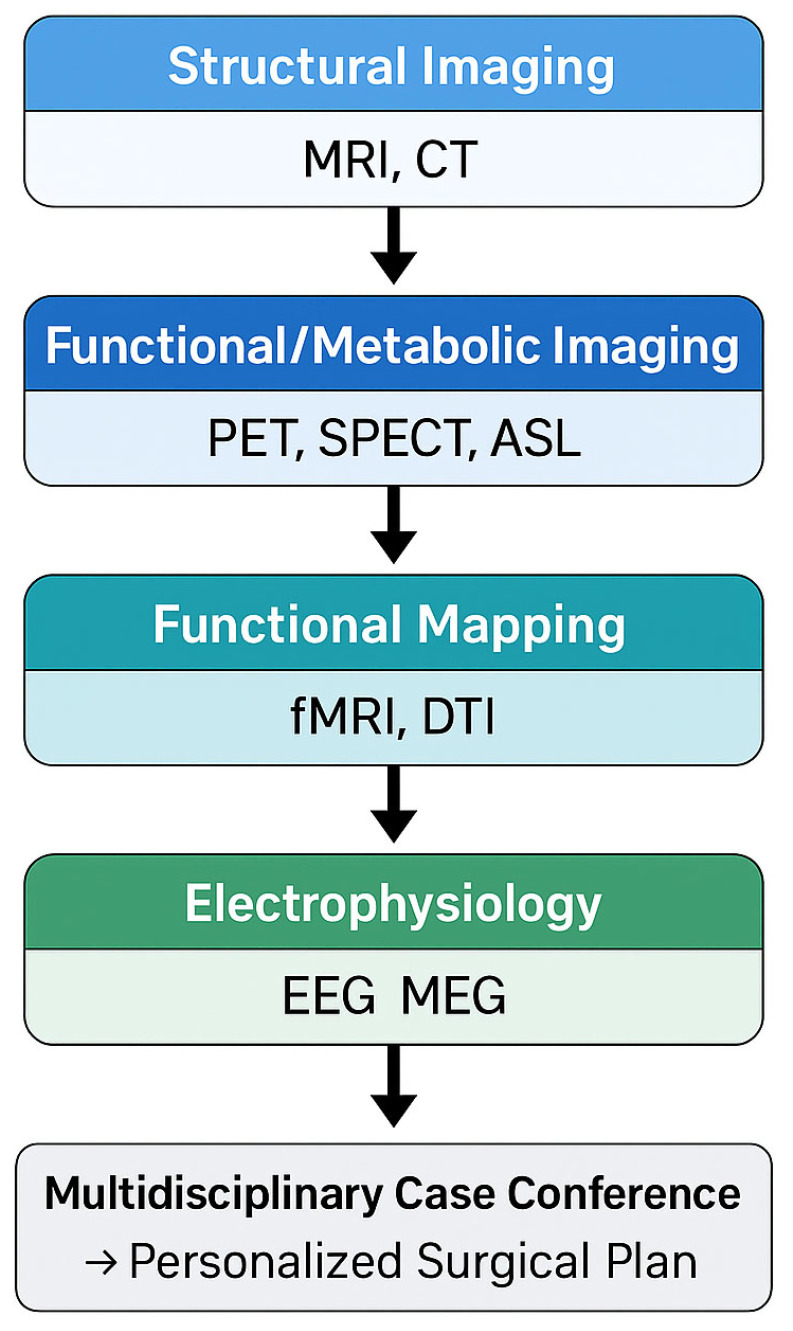
Workflow of Multimodal Imaging Integration.

Innovative imaging modalities and analysis techniques have rapidly evolved in the past decade, enabling clinicians to better localize epileptogenic tissue, delineate its relationship to critical functional areas, and predict surgical outcomes. By combining high-resolution MRI, advanced post-processing algorithms, PET and SPECT functional scans, diffusion tractography, functional MRI mapping, magnetoencephalography (MEG) and electroencephalography (EEG) source localization, the epilepsy surgery team can personalize the treatment strategy for each patient [6]. This review provides a comprehensive overview of how multimodality imaging contributes to personalized neurosurgical planning in epilepsy and can be integrated into a workflow (Figure 1), highlighting current best practices, recent advancements, and future directions. The focus is on maximizing seizure control while minimizing neurological deficits, ultimately presenting the safest and most effective treatment options available and in development for patients with drug-resistant epilepsy.

## 2. Structural Neuroimaging: MRI and Advanced MRI Techniques

MRI is the cornerstone of structural neuroimaging in epilepsy surgery. A high-quality MRI can reveal focal lesions such as hippocampal sclerosis, malformations of cortical development (e.g., focal cortical dysplasia, FCD), tumors, cavernous malformations, gliosis, or encephaloceles that may be the cause of seizures [7]. Identification of such a structural abnormality on MRI is associated with markedly improved surgical outcomes [3]. For example, in temporal lobe epilepsy, an abnormal MRI (such as showing hippocampal atrophy or a tumor) has a positive predictive value of approximately 82% for a good post-surgical outcome, whereas a normal MRI only predicts around 56% chance of success [8]. A systematic review confirmed that the presence of a lesion on MRI or histology raises the odds of seizure freedom 2–3 fold across both temporal and extratemporal lobe surgeries [3]. These findings reinforce that detecting a lesion preoperatively greatly increases confidence that complete resection of the epileptogenic zone is feasible.

### 2.1. Epilepsy-Protocol MRI

To maximize lesion detection, dedicated epilepsy MRI protocols are used at tertiary centers [2]. These typically include high-resolution volumetric T1-weighted sequences, T2 and FLAIR sequences in multiple planes, and sometimes specialized sequences (such as double inversion recovery) to bring out subtle cortical abnormalities [9,10,11]. Scans are often tailored for specific suspected regions (e.g., thin slices through the hippocampus in suspected mesial temporal lobe epilepsy). The International League Against Epilepsy (ILAE) Neuroimaging Task Force recommends a structured “HARNESS-MRI” protocol to ensure consistent high-quality acquisitions for presurgical evaluation [2]. The three core sequences required for optimal lesion detection are (1) a 3-D-T1-weighted sequence (MPRAGE or SPGR) with isotropic voxel size ≤1 mm for precise morphometric analysis; (2) a 3-D-FLAIR sequence with matched resolution to highlight cortical and subcortical gliosis and focal cortical dysplasia; and (3) a high-resolution T2-weighted sequence oriented perpendicular to the long axis of the hippocampus for accurate assessment of mesial temporal sclerosis [2]. Additional recommended sequences include axial and coronal T2, susceptibility-weighted imaging (SWI) to detect cavernomas or microbleeds, and double inversion recovery (DIR) to improve lesion conspicuity in the neocortex [9]. The HARNESS initiative also standardizes 7T MRI acquisition parameters to further enhance contrast-to-noise ratio and detect previously occult epileptogenic lesions [7,11,12]. Together, these sequences represent the minimum dataset for epilepsy-protocol MRI and provide a uniform framework for cross-center comparison and quantitative post-processing.

Despite such protocols, up to 20–30% of cases can still appear “MRI-negative” (no lesion identified) [4]. Many of these patients harbor subtle focal cortical dysplasia or other lesions that elude standard visual detection [10].

### 2.2. MRI Post-Processing

Advanced post-processing techniques have been developed to uncover subtle structural abnormalities in MRI-negative patients. Voxel-based morphometric analysis and other quantitative methods (often referred to as morphometric analysis programs, MAP) can statistically map abnormal brain features (cortical thickness, blurring of gray-white junction, abnormal deep sulci, etc.) by comparing a patient’s MRI to a normative atlas [2,10,13]. These methods effectively enhance the detection of subtle cortical dysplasia, small tumors, or other lesions that are not obvious on conventional radiologist review [2,10,13]. For example, the MAP algorithm has been shown to improve identification of FCD Type II by highlighting areas of abnormally thick cortex or blurred gray-white junction [2]. Implementing MRI post-processing tools can increase the lesion detection rate by roughly 30–50% in MRI-negative epilepsy cases [2,10,13]. In one study, careful re-review of initially negative MRIs with post-processing found previously missed lesions in 27% of patients who had failed an initial epilepsy surgery [2,10,13]. The use of these tools is increasingly recommended as a standard part of presurgical evaluation at comprehensive epilepsy centers [2,10,13]. In fact, prospective studies confirm that incorporating MRI post-processing into routine practice significantly improves lesion detection and should be a routine at tertiary centers [2,10,13].

### 2.3. Ultra-High Field MRI (7T)

Ultra-high field 7 Tesla MRI provides even greater structural resolution and contrast, which can uncover lesions missed on 3T scans [4]. A recent study demonstrated that 7T MRI with advanced post-processing identified epileptogenic lesions in a substantial portion of patients who had normal 3T MRIs [4]. The ability to find these otherwise “invisible” lesions can directly improve surgical targeting and outcomes [4]. Irene Wang et al. (2020) reported that adding 7T MRI led to detecting new lesions in a significant subset of MRI-negative cases, thereby increasing the accuracy of resections and improving seizure outcomes [11]. This is particularly impactful given that up to 40% of surgical candidates have non-lesional 3T MRIs [4]. While 7T MRI is not yet widely available, its use in specialized centers is expanding, and ongoing research (e.g., the HARNESS 7T MRI protocol [2]) indicates that it will play an important role in the future of personalized epilepsy surgery.

### 2.4. Computed Tomography (CT)

CT has a limited role in modern epilepsy surgery workup, as MRI is far superior for visualizing most epileptogenic lesions. However, CT can be useful for detecting calcifications (e.g., in Sturge–Weber syndrome or certain tumors) and is often used intraoperatively or for co-registration with MRI in stereotactic navigation [12]. Historical series relied on CT before the MRI era, but today CT mainly complements MRI in select cases or is fused with MRI for navigational purposes (particularly if MRI is unavailable or contraindicated). In addition, CT plays an important adjunctive role in selected presurgical contexts. High-resolution CT can identify skull defects or bone discontinuities associated with temporal or frontal encephaloceles, which may represent the epileptogenic focus, particularly in MRI-negative temporal lobe epilepsy. CT is also essential during stereoelectroencephalography (SEEG) planning and postoperative verification, allowing precise co-registration of electrode trajectories with MRI datasets to avoid vascular and cortical structures. When fused with MRI, CT provides high-accuracy spatial localization for electrode implantation and resection guidance, complementing the information obtained from structural and functional imaging [12].

State-of-the-art structural imaging, including high-field MRI with dedicated epilepsy protocols and sophisticated post-processing, forms the foundation of a personalized surgical plan. Identifying an anatomical lesion gives a clear target for resection and strongly predicts favorable outcomes [3]. For MRI-negative patients, advanced imaging analysis and 7T scanners are pushing the boundaries to turn “non-lesional” cases into lesional ones, thereby expanding the pool of patients who can benefit from surgery [13].

Below, Section 3 and Section 4 collectively address functional and metabolic neuroimaging modalities used in presurgical evaluation, including PET, SPECT, ASL, and BOLD fMRI. These imaging tools provide complementary physiological information to the structural data obtained by MRI. While neuropsychological and cortical mapping methods are not imaging techniques, they are included to demonstrate how imaging-derived localization data are integrated into multimodal presurgical planning alongside electrophysiological and cognitive assessments.

## 3. Metabolic and Perfusion Imaging: PET, SPECT and ASL

When structural MRI fails to reveal a clear culprit lesion, functional imaging of brain metabolism and perfusion can provide crucial localizing information. These techniques are less spatially detailed than MRI but can highlight regions of abnormal function associated with the epileptic network.

### 3.1. Fluorodeoxyglucose Positron Emission Tomography (FDG-PET)

FDG-PET is a widely used modality in the presurgical evaluation of epilepsy, especially for MRI-negative cases or when multiple abnormalities are present [14,15,16,17]. Interictal FDG-PET scans measure glucose metabolism; epileptogenic brain regions often show hypometabolism interictally due to neuronal dysfunction [15]. In a patient with focal epilepsy, an area of focal hypometabolism on PET that concordantly overlaps with EEG findings can strongly suggest the epileptogenic zone [6]. PET is particularly valuable in temporal lobe epilepsy, with studies reporting sensitivity as high as 80–85% for localizing the seizure focus in MRI-negative temporal lobe cases [16]. Moreover, PET findings can influence surgical decision-making; in cases with normal MRI, showing a focal PET hypometabolism can justify proceeding to invasive EEG monitoring or surgery, whereas a completely normal PET in a non-lesional case might prompt a more cautious approach [17].

For extratemporal lobe epilepsy, PET is still useful but tends to have lower sensitivity and must be interpreted with care (hypometabolic regions might be smaller or multiple). Notably, if seizures are very frequent, interictal PET can occasionally appear normal or even show hypermetabolism due to insufficient interictal period [2]. Despite these caveats, PET often provides critical clues. It can also assist in planning electrode implantation; for instance, guiding where to place depth electrodes in a widespread suspected region by highlighting a metabolic abnormal zone [6]. Hybrid PET/MRI systems are now available, which allow simultaneous acquisition and precise co-registration of metabolic and structural images. Early experiences with PET/MRI suggest an improved yield in detecting subtle lesions by combining metabolic data with high-resolution MRI [18].

### 3.2. Single-Photon Emission Computed Tomography SPECT

Ictal/Interictal SPECT is another functional technique used in many epilepsy centers. SPECT measures cerebral blood flow using injected radiotracers (such as Tc-99m-HMPAO) [19]. In an ictal SPECT, the tracer is injected during or immediately after a seizure, “freezing” a snapshot of cerebral perfusion at the seizure’s peak. The epileptogenic region typically shows hyperperfusion on the ictal scan (due to seizure-related increased blood flow) compared to an interictal baseline scan [6]. By subtracting the interictal from ictal image and co-registering to MRI (a technique known as SISCOM), one can often identify a focal area of dramatically increased perfusion that corresponds to the seizure onset zone [6]. SPECT is especially useful when seizures are infrequent, or MRI/PET are unrevealing [20]. An early meta-analysis in temporal lobe epilepsy showed ictal SPECT could localize the focus in approximately 90–95% of cases when correctly performed, highlighting its power [21]. However, ictal SPECT requires a seizure to be captured with immediate radiotracer injection, which is logistically challenging; it often needs inpatient admission to an epilepsy monitoring unit [22]. Interictal SPECT alone is less useful because interictal perfusion changes can be subtle or absent (except in large lesions or strokes) [23]. Thus, the strength of SPECT lies in ictal-interictal comparison. When successful, SPECT often complements MRI and EEG by pinpointing the region of seizure onset, even when MRI is normal.

### 3.3. Arterial Spin Labeling (ASL)

ASL MRI is an emerging noninvasive perfusion imaging technique that does not require injection of radioactive tracers. ASL uses magnetically labeled blood water as an endogenous tracer to measure cerebral blood flow [24]. In epilepsy, ASL may detect zones of hypoperfusion interictally or hyperperfusion ictally, analogous to SPECT. Recent case reports and series indicate that ASL, especially when processed as z-score maps, can aid in localizing seizure foci in MRI-negative patients [25]. For example, in one illustrative case, an MRI-negative frontal epilepsy, the addition of ASL showed a focal region of hypoperfusion that corresponded to a subtle cortical malformation only apparent after multimodal analysis [2]. ASL can be repeated and is easily included as part of an MRI session, making it attractive for children or others where SPECT/PET are difficult or contraindicated [26]. While ASL is not yet as sensitive as PET or ictal SPECT in routine practice, its role is growing, and it provides another piece of the puzzle in multimodal evaluations [2].

### 3.4. Multimodal Application

In clinical practice, PET and SPECT are often used in complementary fashion. PET (interictal) might highlight an epileptogenic lobe or region, and if more precision is needed, an ictal SPECT with SISCOM can be performed to hone in on the seizure onset zone [6]. These functional scans are interpreted in context with MRI and EEG findings. Notably, functional imaging is far less invasive than intracranial EEG (the gold standard for localization) [6]. They can guide intracranial EEG placement by indicating which brain areas to sample with electrodes [6]. For instance, a patient with non-lesional parietal lobe epilepsy might undergo PET that shows focal hypometabolism in the posterior quadrant, and an ictal SPECT that localizes to the superior parietal lobule; this data can direct a focused implantation of stereo-EEG electrodes to that region, rather than random sampling [27]. Metabolic and perfusion imaging (FDG-PET, SPECT, and ASL) significantly enhance the presurgical workup of epilepsy, particularly for MRI-negative patients. PET can reveal hypometabolic cortical areas suggestive of the epileptogenic zone [6], and SPECT can capture ictal hyperperfusion as a precise localizing indicator [6]. These modalities increase diagnostic confidence and often make the difference in proceeding to surgery or invasive monitoring in cases that would otherwise be equivocal. Multimodal imaging studies consistently show that integrating PET/SPECT with MRI improves the overall detection and characterization of the epileptic focus, thereby facilitating a more personalized and targeted surgical plan [6].

## 4. Functional Mapping with fMRI and Neuropsychological Correlation

Beyond localizing the seizure focus, an equally critical aspect of epilepsy surgery planning is mapping the eloquent cortex (brain regions responsible for key functions like language, memory, motor, and sensory processing) to avoid causing new neurological deficits by the resection. This is where functional MRI (fMRI) and careful neuropsychological assessment come into play in a personalized plan. Functional MRI (fMRI) utilizes blood-oxygen-level-dependent (BOLD) signal changes to map brain activity during specific tasks; in the epilepsy surgery context, fMRI is most commonly used for language and memory mapping, as well as motor and sensory cortex localization [28]. Traditionally, the intracarotid amobarbital procedure (Wada test) was the gold standard for determining language lateralization and memory dominance prior to temporal lobe resections. However, in the last two decades, fMRI has largely supplanted the Wada test in many centers, given its noninvasiveness and adequate accuracy [6]. In fact, in the United Kingdom and other countries, the Wada test is now considered of historical interest only in centers that have fMRI available, because fMRI provides the needed information with far less risk [6]. Language fMRI (e.g., verbal fluency or semantic decision tasks) can reliably lateralize language function (i.e., determine the dominant hemisphere) with a high concordance to Wada results [29]. Studies have shown 80–90% agreement between fMRI and Wada for language lateralization, making it a suitable replacement in most cases [29].

In addition to lateralization, fMRI can map specific language areas (like Broca’s and Wernicke’s) and motor cortex (hand, face, foot areas) by having patients perform tasks during scanning [30]. This information is then used to guide surgical approach and resection boundaries. For example, in a patient with a tumor-related epilepsy near the motor strip, motor task fMRI can identify the hand area, allowing the surgeon to plan a resection that spares this region [31]. Similarly, in temporal lobe epilepsy, fMRI memory paradigms and language tasks can indicate how reliant the patient is on the mesial temporal structures for memory and whether the language cortex is related to the intended resection area [31]. This aids in counseling patients about risks (e.g., memory decline if the dominant hippocampus is resected) and in tailoring the surgical strategy (such as performing a smaller resection or mapping in awake surgery if high risk) [31].

Notably, fMRI has proven useful in predicting and thus minimizing postoperative deficits. Research combining fMRI with direct cortical stimulation (DCS) mapping found that when both methods agree on language-essential areas, the prediction of language outcomes after surgery is quite strong [6]. In one study, fMRI mapping of language had a sensitivity of 80.6% and specificity of 72.7% for identifying critical language sites compared to intraoperative cortical stimulation mapping [6,29,31,32,33]. Moreover, when fMRI and DCS results were concordant, the combination was better at forecasting post-surgical language outcomes than either modality alone [6,29,31,32,33]. This suggests that a well-performed fMRI can in some cases reduce the need for invasive mapping, or at least focus it. Overall, fMRI provides a comprehensive picture of the individual patient’s functional anatomy [6,29,31,32,33]. It helps delineate eloquent cortices that must be preserved, thus personalizing the surgical plan to balance maximal lesion removal with functional safety [6,29,31,32,33]. Indeed, fMRI has been termed a promising alternative to invasive procedures like Wada and even extraoperative cortical stimulation for many mapping purposes [6,29,31,32,33]. It is important to note that fMRI is an indirect measure of neural activity and has limitations. It can sometimes overestimate the extent of functional areas (since BOLD activation might spread beyond essential cortex) [6,29,31,32,33]. Task design and patient compliance are critical, and some patients (e.g., young children or cognitively impaired adults) cannot perform complex tasks in the scanner. In such cases, newer approaches like resting-state fMRI for mapping or passive tasks have been explored, but these are still being validated [32]. Despite limitations, fMRI’s contribution to noninvasive functional mapping is invaluable in formulating a patient-specific surgical approach and reducing morbidity [33].

Neuropsychological evaluation complements imaging by assessing the patient’s baseline cognitive function (memory, language, executive function, etc.) and anticipating how surgery might impact these [34]. While not an imaging modality, neuropsychological testing often correlates with imaging findings (for example, verbal memory impairment might correlate with left hippocampal sclerosis on MRI). These correlations strengthen confidence in the laterality of the seizure focus and also help personalize rehabilitation strategies post-surgery [35]. By combining such assessments with multimodal functional imaging, a tailored surgery that aims not only for seizure control but also for preservation of the patient’s quality of life can be achieved. This personalized risk-benefit analysis, guided by fMRI and cognitive evaluations, is a hallmark of modern epilepsy surgery programs.

## 5. Diffusion Tractography and White Matter Pathway Preservation

While functional MRI focuses on cortical areas, Diffusion Tensor Imaging (DTI) and tractography address the white matter connections between them. In planning an epilepsy surgery, especially for lesions near eloquent pathways, it is crucial to consider the individual’s white matter anatomy [36]. DTI tractography reconstructs the trajectories of major fiber bundles (such as the corticospinal tract, visual radiations, arcuate fasciculus, etc.) by measuring water diffusion directionality in the brain. This enables a personalized 3D map of a patient’s critical white matter tracts, which can be overlaid on their MRI [37]. The use of tractography has become increasingly common in neurosurgical planning for both tumors and epilepsy. For example, in a case of epilepsy due to a lesion in the occipital lobe or posterior temporal lobe, DTI can map the optic radiations (Meyer’s loop) to assess the risk of a visual field deficit (quadrantanopia) if a resection extends too far [38]. In frontal or parietal cases, DTI can map the corticospinal (motor) tracts to avoid causing weakness or paralysis [39]. By integrating these tract maps into nervousness systems, the surgeon can see how close the planned resection is to important pathways and adjust accordingly; this is particularly important in extratemporal epilepsy surgeries, which often involve non-lesional cortex near eloquent areas [40].

Studies have shown that incorporating DTI into preoperative planning can improve functional outcomes. A systematic review of pediatric epilepsy surgery cases found that using DTI tractography was associated with a high rate of favorable postoperative neurological function [38,39,40,41]. In that review of 229 children, multiple reports demonstrated that DTI-informed planning helped avoid motor deficits; in three studies, DTI’s predictions of motor outcome had sensitivities around 80–85% and specificities of 70–100% [38,39,40,41]. Overall, the authors concluded that DTI allows surgeons to create a tailored surgical plan that results in improved functional outcomes, and recommended its routine use in cases at risk for deficits [41]. This aligns with other findings that the risk of postoperative hemiparesis or visual field cuts can be significantly reduced by pre-surgical tract visualization and subsequent preservation of those tracts during resection [38,39,40,41]. Essentially, DTI adds another layer of personalization, not only targeting what tissue to remove (seizure focus) but also highlighting what not to remove or damage (critical white matter highways) [42].

There are also intraoperative uses of diffusion imaging. In some advanced operating rooms, intraoperative MRI or ultrasound with diffusion imaging can update tract location after skull opening or brain shift [43]. Additionally, some systems allow intraoperative tractography where DTI data is used in real-time to navigate (though tractography is usually precomputed). These techniques further ensure that the surgeon stays oriented relative to vital pathways even as brain anatomy changes during surgery [44]. Beyond avoiding deficits, DTI can occasionally aid in localizing the epileptogenic zone indirectly. Certain types of focal cortical dysplasia, for instance, may cause microstructural abnormalities in adjacent white matter that DTI can detect as reduced fractional anisotropy. Changes in structural connectivity patterns (brain networks) have been observed in epilepsy, and in some research settings, network analysis via DTI is used to understand how seizures propagate [45]. However, the main clinical utility of DTI in epilepsy remains risk minimization.

## 6. Integrating Electrophysiology: EEG Source Imaging and MEG

The electrophysiological data from EEG remains a fundamental piece of epilepsy surgery planning. Traditionally, scalp EEG and video monitoring localize the general region of seizure onset, and invasive EEG (such as intracranial electrodes or stereo-EEG) may be used for fine localization when noninvasive methods are insufficient [46]. Modern advances allow the integration of EEG data with imaging, creating a multimodal representation of the epileptic network. EEG Source Imaging (ESI) refers to computational techniques that model the generators of interictal spikes or seizure activity recorded on scalp EEG and project them onto the patient’s MRI (yielding a source localization map) [47]. High-density EEG (with 64–256 electrodes) and sophisticated algorithms can achieve source localization with an accuracy of around 5–10 mm, under ideal conditions [47]. When co-registered with MRI, these localized sources often coincide with the lesion or cortical region later proven to be epileptogenic. ESI is especially helpful in MRI-negative cases or when planning where to place invasive electrodes [47]. For example, if scalp EEG source imaging suggests a focus in the midline frontal cortex, clinicians can target that area with stereo-EEG electrodes even if MRI is normal.

Magnetoencephalography (MEG), on the other hand, is a technique that records the magnetic fields generated by neuronal activity [48]. MEG has the advantage of better spatial resolution than scalp EEG and not being distorted by the skull or scalp, as magnetic fields are less affected by tissue conductivity differences [48]. MEG combined with MRI (to yield magnetic source imaging, MSI) can localize interictal epileptiform discharges with often sub-centimeter precision [49]. Many epilepsy centers perform MEG studies for patients with unclear MRI/EEG findings; the MEG spike localizations are then plotted on the MRI to guide surgical decision-making. Clinical series have shown that resecting the area of clustered MEG spike localizations can lead to good outcomes, and that MEG adds unique information in a significant subset of MRI-negative cases [50]. MEG is particularly sensitive to neocortical epileptic discharges (less so for deep sources), making it useful in cases like neocortical extratemporal lobe epilepsies [51].

**Figure 2:**
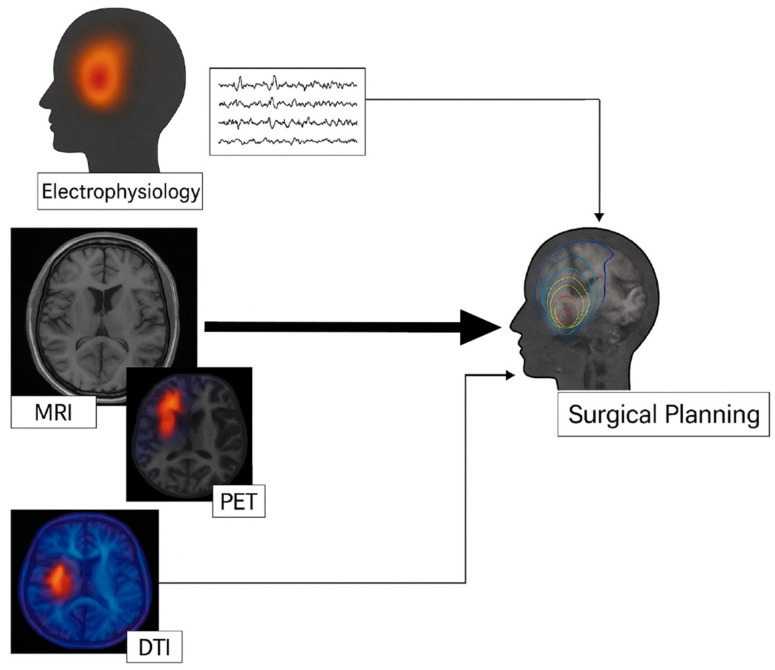
**Schematic representation of multimodal integration in the presurgical evaluation of drug-resistant epilepsy.** Structural MRI and EEG are performed in parallel as first-line studies. Additional functional and metabolic modalities (fMRI, FDG-PET, DTI) provide complementary information that is co-registered and integrated for personalized surgical planning. The arrows represent data convergence rather than temporal order.

Multimodal integration of electrophysiology with imaging is now strongly advocated. One ILAE Neuroimaging Task Force report emphasized that integrating multimodal imaging and electrophysiology data in the same 3D space allows accurate analysis of their relationships [2]. By co-registering MRI (structural lesion), PET/SPECT (functional changes), and MEG or EEG source localizations (electrical activity) all together, the concordance or discrepancies become visually apparent [52]. This can increase the diagnostic confidence, especially when findings are subtle or conflicting [2]. For instance, suppose an MRI is normal, PET shows hypometabolism in the right frontal region, and MEG shows clustered spike sources in the same area, the overlay of these on one brain model provides compelling evidence of a right frontal epileptogenic zone, justifying invasive monitoring or resection in that area (Figure 2). If, on the other hand, the MEG clusters in a different lobe than the PET, it signals a need for broader investigation. At many comprehensive epilepsy centers, a multidisciplinary case conference is held where neurologists, neurosurgeons, radiologists and others review all data, MRI, PET, SPECT, EEG, MEG, and neuropsychology, often using specialized software to display co-registered results. This integrated review is essential for formulating the personalized surgical plan for each patient [5]. In the past, physicians had to mentally fuse this information, but now dedicated platforms (like Curry, Brainstorm, EpiNav or custom hospital systems) allow true multimodal integration for visualization [5]. The Cleveland Clinic, for example, reported on their multimodal image integration (MMII) workflow where all available imaging and EEG data are merged; among 467 patients who underwent this MMII process, including many with prior failed surgeries or MRI-negative epilepsy, the information was invaluable for electrode planning and resection strategy [5]. In that cohort, about 56% of patients were MRI-negative, yet after integration and tailored invasive studies, 50% achieved complete seizure freedom one year post-surgery [5]. This illustrates how critical the proper addition of multimodal data is in achieving surgical success in challenging cases. Even postoperative imaging can be integrated with preoperative data to evaluate surgical results. By loading a post-resection MRI or CT scan into the same space as pre-op PET, MEG, etc., one can verify if the resection indeed covered all the identified hotspots (and if not, it might explain a failure) [2]. This approach is useful in re-operation planning for those who did not become seizure-free as it helps identify residual epileptogenic tissue that was missed [2].

To summarize the range and complementarity of imaging methods described above, Table 1 summarizes the principal neuroimaging modalities currently employed in presurgical evaluation of drug-resistant epilepsy. The table delineates for each technique the nature of information obtained such as structural, metabolic, perfusion, or functional, as well as its main clinical applications and inherent limitations. This comparative overview highlights how combining multiple modalities enables a comprehensive characterization of the epileptogenic zone and its relationship to eloquent cortex, thereby informing a more precise and individualized surgical plan.

## 7. Multimodal Image-Guided Surgery and Technological Innovations

With the array of preoperative imaging modalities now available, a major challenge and opportunity is how to utilize all this information during surgery. The operating room has seen significant technological innovations that allow surgeons to bring multimodal data to bear in real time, further personalizing the surgical execution.

### 7.1. Neuronavigational Systems and Intraoperative Visualization

These are computer systems (often with stereotactic infrared cameras) that map the patient’s imaging data to their physical head during surgery. Traditionally, neuronavigation uses the structural MRI (and CT if needed) to guide the surgeon’s tools to the target [53]. Modern neuronavigation, however, can incorporate multimodal data; for instance, the surgeon can see the MRI with an overlay of PET hypometabolic zone, MEG spike focus, and DTI tracts, all co-registered in the navigation view [5]. If the patient underwent stereo-EEG, the electrode contact locations (visible on CT) can also be marked on the MRI and displayed. This means the surgeon has a 3D roadmap of not just anatomy but also functional and electrophysiological landmarks. Jin et al. (2021) described how such multimodal image integration was employed in practice, noting that it was especially helpful for planning stereo-EEG trajectories and tailoring resection extent [5]. By inputting all data into a unified platform (e.g., the Curry software or similar), they could virtually plan the surgery and then use navigation to execute it [5].

One exciting advance in intraoperative visualization is the use of augmented reality (AR) in the operating microscope or surgeon’s eyepiece. In AR, the multimodal imaging maps are superimposed onto the surgeon’s view of the actual brain [54]. For example, at the Medical University of Vienna, surgeons conducted a study on pediatric focal epilepsy cases using an AR-equipped surgical microscope [38]. They loaded functional neuronavigational data (fMRI language areas, DTI tracts, PET/SPECT findings, and even the outline of stereo-EEG-defined epileptic tissue) as 3D reconstructions, and AR allowed these to be projected onto the real anatomy during microsurgery [38]. This greatly helped in delineating the tissue to resect versus to spare. In 43 pediatric epilepsy surgeries using this multimodal AR guidance, 83% of patients who had at least one-year follow-up achieved a favorable seizure outcome (ILAE Class 1, meaning seizure-free or only auras) [38]. Even among the 25% of patients who were MRI-negative and required invasive monitoring first, AR facilitated complete resection of the EEG-defined focus [38]. Importantly, the morbidity was low, a transient hemiparesis in 14% and a quadrantanopia in 7%, with no permanent new deficits reported [38]. The authors concluded that AR-supported resection is a promising tool to target both lesional and non-lesional epileptogenic zones with high precision and safety [38]. This technology essentially brings the preoperative multimodal plan to life in the OR, enhancing the surgeon’s spatial awareness. Similarly, virtual reality (VR) planning tools are being used preoperatively. Platforms such as Surgical Theater^®^ convert 2D scans (MRI, CT, PET, etc.) into immersive 3D models that surgeons (and even patients) can “fly through” in virtual reality [55]. This helps in understanding complex anatomies and planning approaches. While VR is usually a planning aid (before surgery), AR is used during surgery. Both serve the goal of making the vast multimodal data intuitively understandable to the surgeon, thus increasing the likelihood of a tailored and effective resection [56].

### 7.2. Intraoperative Adjuncts

Intraoperative MRI and ultrasound allow surgeons to verify and update imaging during the operation, which is particularly useful if brain shift occurs or to check extent of resection. In epilepsy surgery, intraoperative MRI can confirm that the lesion or focus (e.g., a subtle cortical dysplasia) has been completely removed before closing, which in a personalized sense ensures the surgical goal was met [57]. Intraoperative electrocorticography (ECoG), while not imaging per se, is often used to guide resection margins by recording EEG directly from the cortex after the lesion removal; persistent epileptic discharges might prompt additional resection [58]. All these tools (ECoG, imaging, functional mapping in awake cases) can be combined intraoperatively for maximal effect.

### 7.3. Laser Interstitial Thermal Therapy (LITT)

This is a minimally invasive surgical alternative for certain epilepsy cases (such as mesial temporal lobe epilepsy or small hypothalamic hamartomas) where a laser fiber is inserted into the lesion and heats it to ablate it, under MRI guidance [59]. While not an imaging modality, LITT’s rise is part of personalized therapy; often chosen for patients with deep lesions or those at high risk from open surgery [60]. Imaging is crucial in LITT, real-time MRI thermography guides the ablation to ensure the target is destroyed and surrounding tissue is spared. The selection of LITT vs. open resection for a patient is based on multiple factors, including imaging features like lesion size, location, and relationship to critical structures (for example, a small amygdala/hippocampus focus might be amenable to LITT, whereas a diffuse dysplasia might require open surgery) [61]. Thus, advanced imaging indirectly influences which surgical modality is chosen for each patient.

### 7.4. Robotics and Navigation

Stereotactic robots for electrode placement (in SEEG) or for LITT fiber insertion are increasingly used. They rely on the preoperative multimodal plan (with all imaging integrated) to execute precise trajectories; this improves safety (for example, avoiding vessels seen on vascular imaging or avoiding tracts seen on DTI) and accuracy of targeting the epileptic focus [62]. High accuracy electrode implantation then yields better recordings, which feeds back into identifying the surgical target [62]. Overall, these technological innovations underscore the current trend: the convergence of data and delivery. We now have the ability to not only gather multimodal information preoperatively but to seamlessly incorporate it into the surgical workflow. As these technologies mature, we can expect even greater precision and better outcomes.

## 8. Emerging Directions and Future Perspectives

The landscape of neuroimaging and personalized neurosurgery for epilepsy continues to evolve rapidly. Some emerging and future directions include artificial intelligence (AI) and machine learning (ML) that are being harnessed to analyze complex multimodal datasets and detect subtle patterns that elude human observers. A prime example is the Multicentre Epilepsy Lesion Detection (MELD) project, which developed a machine learning algorithm to identify focal cortical dysplasias on MRI [63]. By training on over a thousand MRIs from 22 epilepsy centers, the MELD AI was able to find lesions in 67% of cases overall, and remarkably, it detected the previously “hidden” FCD in about 63% of patients whose MRIs had been reported as normal by experts [64]. In other words, AI can substantially improve the yield of MRI, enabling many patients with negative scans to finally receive a diagnosis and be considered for curative surgery [65]. Moreover, the MELD tool was designed to be interpretable, highlighting specific cortical features (thickness, surface area, etc.) that are abnormal [64]. This transparency helps clinicians trust and adopt the tool. Beyond lesion detection, machine learning models are being explored for predicting surgical outcomes based on multimodal inputs (imaging, EEG, clinical data). Early studies show promise in using ML to prognosticate which patients will become seizure-free [66]. In the near future, AI might assist the multidisciplinary team by aggregating all the patient’s data and suggesting the optimal individualized plan, or even alerting to discrepancies (for instance, if an imaging finding does not align with EEG, an algorithm might flag it for further review).

There is a paradigm shift from viewing epilepsy as a single focus to considering it as a network disorder in many cases. Advanced imaging like resting-state fMRI, diffusion connectomics, and MEG/EEG connectivity analyses allow mapping of the epileptic network, identifying not just the core “node” to remove but also the supportive nodes and pathways that might need modulation [67]. Personalized connectome analysis could influence surgery by identifying, for example, that two distant lesions are part of one network (necessitating dual surgery), or that a single lesion triggers network hyperexcitability (suggesting a smaller resection plus neuromodulation might suffice) [67]. Some researchers are using graph theory on DTI or EEG-fMRI data to pinpoint influential network hubs of seizures [5]. In the future, the surgical approach might involve multitarget strategies (resect one node, ablate another, stimulate a third) tailored to the patient’s unique network; a truly personalized approach beyond one-lesion one-resection.

Furthermore, as 7T MRI becomes more accessible, even higher field strengths (11.7T for research) and new MRI sequences (e.g., quantitative T1 mapping, susceptibility imaging for microstructural changes) will further enhance lesion detectability. Magnetic resonance spectroscopy (MRS) can noninvasively detect metabolic abnormalities (like decreased N-acetylaspartate or increased choline) in focal epilepsy and might localize a focus or grade the tissue’s health [68]. PET tracers beyond FDG are also being investigated; for instance, ^11C-Flumazenil PET can bind to GABA-A receptors and potentially highlight epileptogenic cortex (since such cortex may have reduced receptor binding) [69]. New tracers for inflammation (TSPO PET) or epileptogenesis (like probes for neuroinflammation or glutamate receptors) could add another dimension to preoperative evaluation in coming years [70]. These advances, combined with AI to interpret them, will make the imaging evaluation even more comprehensive. A big focus, as pointed out by experts, is to make these advanced multimodal techniques widely available, not just at a few elite centers [2]. Efforts like open-source software (e.g., the released SWANe pipeline for multimodal imaging analysis [2]) and training programs for clinicians aim to disseminate these capabilities globally. This is important for equity, so that patients with intractable epilepsy anywhere can benefit from a state-of-the-art personalized workup and not be deemed inoperable just for lack of advanced imaging.

Finally, when a complete resection is not possible (due to eloquence or multifocal disease), personalized neurosurgery might entail devices like responsive neurostimulation (RNS) or deep brain stimulation (DBS) targeting the specific network of that patient’s epilepsy [71]. Imaging is crucial here too, as placing a DBS electrode in the anterior nucleus of the thalamus requires MRI guidance, or targeting an RNS lead to a small lesion seen only on high-res MRI [72]. The decision of which patients receive resection vs. neuromodulation will be aided by imaging and likely by ML models that incorporate imaging findings to predict who is a good surgical vs. device candidate [73]. The future will likely see even tighter collaboration between disciplines (e.g., computer scientists embedded in epilepsy teams to run AI analyses, engineers developing better AR/VR for surgeons, etc.); the complexity of multimodal data demands such teamwork.

## 9. Conclusions

Epilepsy surgery has entered an era where personalization is the defining paradigm. No longer is surgical planning based on a single MRI or a single EEG finding; instead, it synthesizes a wealth of information about the patient’s brain structure, function, and network into a tailored treatment strategy. The integration of multimodality imaging: structural MRI (often enhanced by post-processing and higher field strength), metabolic PET scans, perfusion SPECT/ASL, functional MRI maps, diffusion tractography, and electrophysiological source localization, provides a 360-degree evaluation of each patient. As we have reviewed, leveraging these modalities in concert increases the likelihood of pinpointing the epileptogenic zone (even in cases previously labeled “non-lesional”), while also charting the safest path for intervention. Multimodal approaches can uncover subtle lesions, improve diagnostic confidence, and expand surgical candidacy to more patients, all while minimizing risks. Personalized imaging allows risk stratification and patient counseling that is far superior to earlier eras of epilepsy surgery. Looking ahead, we can expect the gap between preoperative virtual planning and intraoperative reality to close even further, with technologies like augmented reality, robotics, and AI-driven guidance becoming routine tools for the neurosurgeon. Continued research (both preclinical and clinical) is necessary to refine these tools and validate emerging ones. The ultimate vision is that every person with drug-resistant epilepsy, regardless of whether they have an obvious lesion, will receive a thorough, cutting-edge evaluation that leaves no stone unturned, so that if they are a surgical candidate, the operation is planned and performed with as much knowledge and precision as possible. The rapidly evolving landscape of neuro-oncology and neurosurgery, as exemplified by precision tumor targeting and in epilepsy by the strategies detailed herein, points to a future where personalized brain surgery is not just an aspiration but the standard of care. The knowledge compiled in this review serves as a valuable resource for healthcare professionals and researchers to remain updated of these advances and to continue pushing the boundaries of what is possible in caring for patients with epilepsy.

## Figures and Tables

**Table 1:** Summary of neuroimaging modalities used in presurgical evaluation of drug-resistant epilepsy.

Imaging Modality	Type of Finding	Main Strengths/Clinical Utility	Limitations
MRI (3 T/7 T)	Structural anatomy	Detects hippocampal sclerosis, FCD, tumors, cavernomas; provides high spatial resolution; foundation of presurgical planning	May miss subtle FCD or microdysplasia; requires expert interpretation
Post-processed MRI (MAP, VBM)	Structural/morphometric	Enhances detection of subtle cortical abnormalities in MRI-negative cases	Dependent on normative database and post-processing expertise
CT	Bone/calcification	Detects calcified lesions, skull defects, or encephaloceles; essential for SEEG planning and co-registration	Limited soft-tissue contrast; radiation exposure
FDG-PET	Metabolic (glucose)	Identifies interictal hypometabolism concordant with epileptogenic zone; useful in MRI-negative or multifocal cases	Low spatial resolution; interictal variability; radiation exposure
Ictal/Interictal SPECT (SISCOM)	Perfusion	Captures ictal hyperperfusion for precise seizure-onset localization	Requires seizure capture and rapid tracer injection; limited availability
ASL-MRI	Perfusion (non-radioactive)	Detects hypo-/hyper-perfusion without contrast or radiation; useful in children	Lower sensitivity than PET/SPECT; still emerging in routine practice
fMRI (BOLD)	Functional activation	Non-invasive mapping of language, motor, memory networks; replaces Wada test in many centers	Task-dependent; motion and compliance sensitive; indirect neuronal measure
DTI/Tractography	White-matter connectivity	Maps eloquent tracts (e.g., corticospinal, arcuate); guides resection margins	Susceptible to distortion; limited near lesions or edema
EEG Source Imaging (ESI)	Electrophysiological	Non-invasive source localization integrated with MRI	Spatial accuracy limited by electrode density and head model
MEG/MSI	Electrophysiological (magnetic)	High temporal and spatial precision; helpful in MRI-negative and neocortical cases	Expensive; limited sensitivity for deep foci

## Data Availability

No new data were created or analyzed in this study. Data sharing is not applicable to this article.
